# Gut microbiome association with brain imaging markers, APOE genotype, calcium and vegetable intakes, and obesity in healthy aging adults

**DOI:** 10.3389/fnagi.2023.1227203

**Published:** 2023-09-06

**Authors:** Tyler C. Hammond, Stefan J. Green, Yael Jacobs, George E. Chlipala, Xin Xing, Sally Heil, Anna Chen, Chetan Aware, Abeoseh Flemister, Arnold Stromberg, Priti Balchandani, Ai-Ling Lin

**Affiliations:** ^1^Sanders-Brown Center on Aging, University of Kentucky, Lexington, KY, United States; ^2^Department of Neuroscience, University of Kentucky, Lexington, KY, United States; ^3^Genomics and Microbiome Core Facility, Rush University, Chicago, IL, United States; ^4^BioMedical Engineering and Imaging Institute, Icahn School of Medicine at Mount Sinai, New York, NY, United States; ^5^Research Informatics Core, University of Illinois Chicago, Chicago, IL, United States; ^6^Department of Computer Science, University of Kentucky, Lexington, KY, United States; ^7^Roy Blunt NextGen Precision Health, University of Missouri, Columbia, MO, United States; ^8^Department of Radiology, University of Missouri, Columbia, MO, United States; ^9^School of Medicine, University of Missouri, Columbia, MO, United States; ^10^Dr. Bing Zhang Department of Statistics, University of Kentucky, Lexington, KY, United States; ^11^Institute for Data Science and Informatics, University of Missouri, Columbia, MO, United States; ^12^Division of Biological Sciences, University of Missouri, Columbia, MO, United States

**Keywords:** healthy aging, microbiome, MRI, APOE, BMI, diabetes, calcium intake, brain metabolism

## Abstract

**Introduction:**

Advanced age is a significant factor in changes to brain physiology and cognitive functions. Recent research has highlighted the critical role of the gut microbiome in modulating brain functions during aging, which can be influenced by various factors such as apolipoprotein E (APOE) genetic variance, body mass index (BMI), diabetes, and dietary intake. However, the associations between the gut microbiome and these factors, as well as brain structural, vascular, and metabolic imaging markers, have not been well explored.

**Methods:**

We recruited 30 community dwelling older adults between age 55-85 in Kentucky. We collected the medical history from the electronic health record as well as the Dietary Screener Questionnaire. We performed APOE genotyping with an oral swab, gut microbiome analysis using metagenomics sequencing, and brain structural, vascular, and metabolic imaging using MRI.

**Results:**

Individuals with APOE e2 and APOE e4 genotypes had distinct microbiota composition, and higher level of pro-inflammatory microbiota were associated higher BMI and diabetes. In contrast, calcium- and vegetable-rich diets were associated with microbiota that produced short chain fatty acids leading to an anti-inflammatory state. We also found that important gut microbial butyrate producers were correlated with the volume of the thalamus and corpus callosum, which are regions of the brain responsible for relaying and processing information. Additionally, putative proinflammatory species were negatively correlated with GABA production, an inhibitory neurotransmitter. Furthermore, we observed that the relative abundance of bacteria from the family *Eggerthellaceae*, equol producers, was correlated with white matter integrity in tracts connecting the brain regions related to language, memory, and learning.

**Discussion:**

These findings highlight the importance of gut microbiome association with brain health in aging population and could have important implications aimed at optimizing healthy brain aging through precision prebiotic, probiotic or dietary interventions.

## Introduction

Aging is associated with significant alterations in brain structure, blood flow, and metabolism, leading to declines in cognitive function and increased risk for neurodegenerative diseases. The most notable structural change is brain atrophy, particularly in the hippocampus and other regions associated with memory and cognitive processes (Raz and Rodrigue, 2006; Fjell and Walhovd, 2010). Aging also leads to a decline in white matter (WM) integrity through various processes, including myelin degradation, and the development of white matter lesions (Molloy et al., 2021). Cerebral blood flow (CBF) is another critical aspect of brain function that changes with age. Older adults typically exhibit reduced CBF, which can negatively impact cognition (Tarumi and Zhang, 2018). Aging also affects brain metabolism, with older individuals displaying reduced glucose and oxygen metabolic rates, and the production of essential brain metabolites (Lin et al., 2014; Brinton et al., 2015; Mattson and Arumugam, 2018; Turner et al., 2022).

Accumulating evidence has implicated the gut microbiome in brain health (Cox and Weiner, 2018). Gut dysbiosis is associated with many neurological disorders, including Alzheimer’s disease, stroke, and traumatic brain injury (Vogt et al., 2017; Borsom et al., 2020; Yanckello et al., 2022a; Hammond et al., 2022b). Aging is associated with significant alterations in the gut microbiome (Hoffman et al., 2017). One prominent age-related change is the decline of beneficial bacteria, such as *Bifidobacteria*, and an increase in opportunistic pathogens like bacteria from the phylum *Proteobacteria* (Biagi et al., 2010). This shift may contribute to chronic low-grade inflammation, known as “inflammaging,” which has been linked to various age-related conditions, including cardiovascular diseases, cognitive decline, and metabolic disorders (Franceschi and Campisi, 2014). Additionally, older adults often experience reduced production of short-chain fatty acids (SCFAs) due to changes in the gut microbiota (Badal et al., 2020). SCFAs play essential roles in maintaining gut health, immune function, and metabolism. The decline in SCFAs production may contribute to age-related immune dysregulation and increases susceptibility to infections and chronic diseases (Buford, 2017).

There are other factors that may impact gut-brain interactions in aging, such as apolipoprotein E (APOE) variance, diet, body mass index (BMI), obesity, and diabetes. Individuals with APOE ε4 alleles (APOE4) display altered brain function, such as reduced amyloid-beta clearance (Castellano et al., 2011) and increased inflammation (Shi et al., 2017). Recent studies show connections between the APOE4 genotype and gut microbiome composition, with APOE4 carriers exhibiting increased pro-inflammatory microbes (Hoffman et al., 2019; Tran et al., 2019; Parikh et al., 2020; Yanckello et al., 2022b). Diet, BMI, and diabetes all have significant impacts on brain function and the gut microbiome (Asadi et al., 2022; Wachsmuth et al., 2022). A diet high in sugar and saturated fat can contribute to inflammation in the brain, impairing cognitive function and increasing the risk of dementia (Gómez-Pinilla, 2008). Additionally, obesity and diabetes are associated with decreased cognitive performance, and an altered gut microbiome, which can contribute to inflammation and neurodegenerative diseases (Arnoldussen et al., 2014; Gomes et al., 2014).

While there is growing evidence to support a link between the gut microbiome and brain health in aging, the specific associations between microbiome composition, brain imaging features, APOE genotype, BMI, diabetes, and dietary intake remain unclear. To shed light on these relationships, we performed an analysis of 30 community-swelling older adults. Our study aims to establish a foundation for understanding how gut microbes are associated with brain health. These findings could have important implications for developing precision nutrition interventions to optimize healthy brain aging older adults.

## Materials and methods

### Participants

We recruited participants for this study in Kentucky, United States. The inclusion criteria for the study required that participants be between age 55–85. Exclusion criteria required that participants not have an acute disease of chronic, clinically significant (unresolved, requiring on-going medical management or medication) pulmonary, gastrointestinal, dermatologic, hepatic, or renal functional abnormality. Participants were required to not have had cancer or a positive test for HIV, HBV, or HCV. Participants were required to not be immunosuppressed or have had major surgery of the GI tract in the past 5 years. Participants also had to be MRI compatible. The sample size was determined based on the relative abundance comparisons of the gut microbiome with the highly specific species level resolution provided by whole genome sequencing technology. With alpha = 0.05, a sample size of 30 and continuous variables, we will have 80% power to detect Pearson correlation coefficient ρ of 50% or more. Although the pandemic has imposed challenges for recruitment and retention, especially for the aging population, we were able to complete the study with 30 participants by taking into consideration the population distribution of Kentucky – 85.08% White Non-Hispanic and 7.88% Black or African American Non-Hispanic (Source: US Census 2017 5-Year American Community Survey). Participants were recruited through researchmatch.org and from advertisements posted by the Center for Clinical and Translational Science at the University of Kentucky. Table 1 describes the major demographic characteristics of our participants known to influence brain aging, with a mean age of 65.7 years, 17.23 years of education, and BMI of 26.95 kg/m^2^. Our population was 83.33% female, 90% white, 10% had diabetes, 43.33% had hypertension, and 30% had hyperlipidemia. 53% were genotype APOE ε3/ε3, 33% were ε4 carriers, and 13% were ε2 carriers. Participants were required to not all research activities were monitored by the Institutional Review Board at the University of Kentucky.

**Table 1:** Participant characteristics.

Participants	*N* = 30
Age	65.7 ± 5.79
Sex (% Female)	83.33%
Race (% White)	90.00%
(% Black)	6.67%
(% Asian)	3.33%
Genotype (% APOE ε3/ε3)	53.33%
(% APOE ε3/ε4)	30.00%
(% APOE ε4/ε4)	3.33%
(% APOE ε2/ε3)	13.33%
Education	17.23 ± 1.80
BMI	26.95 ± 5.36
Diabetes	10.00%
Hypertension	43.33%
Hyperlipidemia	30.00%

### Study design

Each individual received a verbal and written explanation of the purposes, procedures, and potential hazards of the study, and written consent was obtained. Study personnel consented subjects using the University of California, San Diego Brief Assessment of Capacity to Consent (UBACC) to ensure capacity. Signed consent was obtained before definite enrollment of the subject in this study. Following consent, we obtained past medical history from the electronic health record and questionnaires. These data were used solely for research purposes. Variables including age, gender, racial/ethnic background, education level, Body Mass Index, history of conventional vascular risk factors (hypertension, diabetes mellitus, atrial fibrillation, hyperlipoproteinemia, and smoking habit), pre-stroke therapy, and acute treatment (i.e., oral anticoagulants, antiplatelet agents, tPA, IV thrombolysis and/or mechanical thrombectomy, and/or antibiotics) were recorded and used as covariates for our analyses.

### Stool sample collection and analysis

Stool samples were collected in Zymo DNA stabilization solution with Sarstedt feces tubes from feces catcher placed on toilet seat. Genomic DNA was extracted from 0.25 grams of stool using ZymoBIOMICS^™^ DNA Mini Kit and shipped to the Genomics and Microbiome Core Facility (GMCF) at Rush University. Shotgun metagenome libraries were constructed using an Illumina DNA prep kit according to the manufacturer’s instructions. Deep sequencing of libraries was performed on an Illumina NovaSeq6000 instrument, using paired end 2 × 150 base sequencing reads. Unassembled sequencing reads were analyzed by the Research Informatics Core at the University of Illinois Chicago for microbiome analysis. We used MetaPhlAn (Metagenomic Phylogenetic Analysis) to profile the composition of microbial communities using unique clade-specific marker genes identified from ~17,000 reference genomes (~13,500 bacterial and archaeal, ~3,500 viral, and ~ 110 eukaryotic) (Beghini et al., 2021).

### Diet analysis

We assessed diet history using the Dietary Screener Questionnaire in the National Health and Nutrition Examination Survey (DSQ) (NHANES 2009-10). DSQ was used to measure dietary intake over the last month to include estimated intake of fiber, calcium, whole grains, sugar, dairy, fruits and vegetables, and sugar sweetened beverages.

### APOE genotyping

We collected oral swabs from all participants and placed them in Zymo DNA stabilization solution. We sent the oral swabs to the Research Informatics Core at the University of Illinois Chicago for DNA extraction and amplification. The Core performed PCR to amplify and measure SNPs rs429358 and rs7412 that define the common allelic variants of Apolipoprotein E.

### Imaging acquisition

MRI images were collected from all participants on a 3 T Prisma MR scanner (Siemens, Germany) at UK’s Magnetic Resonance Imaging & Spectroscopy Center.

#### Structural imaging

High-resolution, 3D anatomic images were acquired using an MP-RAGE sequence [repetition time (TR) = 2,530 ms, echo time (TE) = 2.26 ms, flip angle (FA) = 7°, 1 mm isotropic voxels, 6:19 min]. We used the FreeSurfer Software Suite to segment and quantify brain volumes.

#### Magnetic resonance spectroscopy

Brain metabolites were measured with a chemical shift imaging (CSI) sequence that incorporates localization by adiabatic selective refocusing (LASER) for FOV-reduction (McNab and Bartha, 2006). Semi-laser sequence [TR = 1700 ms and TE = 40 ms]. All MRS slices were placed parallel to the anterior commissure-posterior commissure line. The volume of interest was centered to the medial to posterior part of the corpus callosum, with VOI = 80 (l-r) × 80 (a-p) and field of view (FOV) = 160 × 160 mm^3^. The acquired matrix size was 10 × 10 × 15 mm. Two voxels that contained pure white matter were selected from each participant and averaged for analysis. Spectra were calculated using LCModel Software (Provencher, 1993) to determine the concentration of brain metabolites.

#### Arterial spin labeling

Quantitative CBF (with units of mL/g per minute) was measured using a pulsed arterial spin labeling (PASL) PICORE Q2T sequence with a TR = 4,400 ms and a TE = 20.8 ms. We used FreeSurfer software to process relative cerebral blood flow (relCBF) data produced by the Siemens scanner.^1^ The automated software provides relCBF averages over the FreeSurferColorLUT.txt set of ROIs.

#### Diffusion tensor imaging

White matter integrity was measured using an axial double refocused spin echo, echo planar imaging (Yang et al., 2015) Diffusion Tensor Imaging (DTI) sequence with the following parameters: TR = 3,400 ms, TE = 71 ms, field of view = 232 mm, 81 slices, 2 mm isotropic resolution. The DTI images were acquired with 128 noncollinear encoding directions (*b* = 2000 s/mm^2^) and 6 images without diffusion weighting (*b* = 0 s/mm^2^, b0). DTI data were analyzed with FSL (Functional MRI of the Brain software library, FMRIB) to calculate fractional anisotropy (FA) values.

### Statistical analyses

All statistical analyses were completed using JMP Statistical Software (SAS, Cary, NC, USA) and R Statistical Software (R Core Team, 2020). Relative abundance comparisons were utilized to provide detailed analyses of the samples concordant with the highly specific species level resolution provided by whole genome sequencing technology. Shapiro-Wilks testing revealed that there were no significant departures from normality. For all comparisons, Two-sample *t*-test and two-way ANOVA were used to determine differences between groups with a continuous response. The MaAsLin2 R Package was used to normalize all variables and employ linear regression analysis to associate various variables with microbiome measures (Mallick et al., 2021). A false discovery rate (FDR) of *q* < 0.25 was used as part of our exploratory approach of identifying differentially abundant microbial features due to a small dataset. With alpha = 0.05, a sample size of 30 and continuous variables, we will have 80% power to detect Pearson correlation coefficient ρ of 50% or more.

## Results

### Gut microbiome associations with APOE genotype, BMI, diabetes, and hypertension

**Figure 1:**
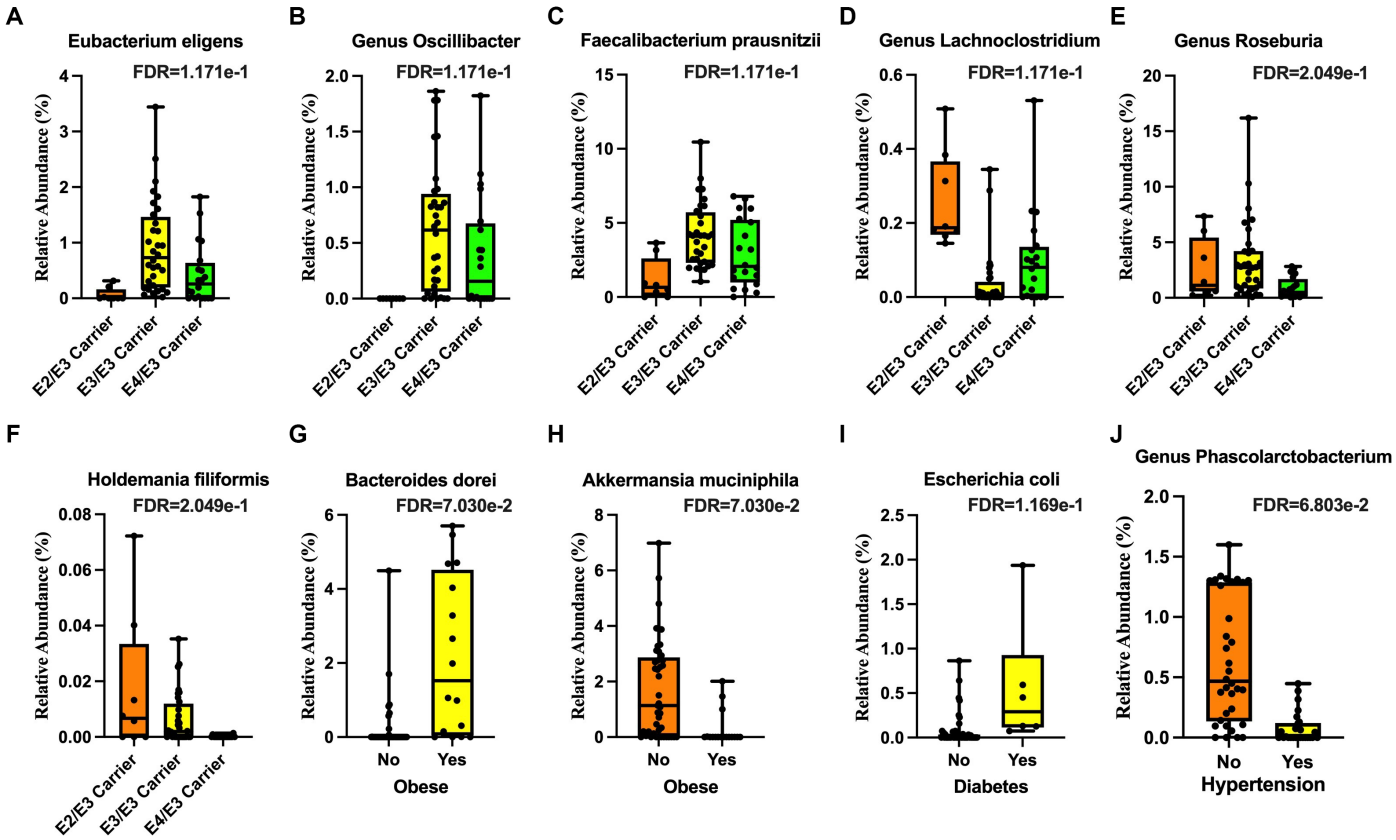
Microbial taxa associated with demographic features. The Apolipoprotein E (APOE) ε2 genotype was associated with lower **(A)** *Eubacterium eligens*, **(B)** the genus *Oscillibacter*, and **(C)** *Faecalibacterium prausnitzii* and higher **(D)** genus *Lachnoclostridium*. The APOE ε4 genotype was associated with lower **(E)** genus *Roseburia* and **(F)** *Holdemania filiformis*. Obesity was associated with higher **(G)** *Bacteroides dorei* and lower **(H)** *Akkermansia muciniphila*. Diabetes was associated with higher **(I)** *Escherichia coli* (*E. coli*). Hypertension was associated with lower **(J)** genus *Phascolarctobacterium*.

We detected significant associations between gut microbial community structure with APOE genotype, BMI, diabetes, and hypertension in our community dwelling older adult cohort. The relative abundance of bacterial species from the phylum Firmicutes, including *Eubacterium eligens* (Figure 1A), *Oscillibacter* (Figure 1B), and *Faecalibacterium prausnitzii* (Figure 1C), were all lower in the APOE ε2/ε3 (APOE2 carriers) genotype and that of the genus *Lachnoclostridium* was higher (Figure 1D). In the APOE ε3/ε4 (APOE4 carriers) genotype, the relative abundance of *Roseburia* (Figure 1E) and *Holdemania filiformis* (Figure 1F), both from the phylum Firmicutes, was lower. The relative abundance of *Bacteroides dorei* (phylum Bacteroidetes) was higher (Figure 1G), while that of *Akkermansia muciniphila* (phylum Verrucomicrobia) was lower in obesity (Figure 1H). The relative abundance of *Escherichia coli* (*E. coli*; phylum Proteobacteria) was higher in participants with diabetes (Figure 1I). The relative abundance of *Phascolarctobacterium* (phylum Firmicutes) was lower in participants with hypertension (Figure 1J).

### Gut microbiome associations with calcium and vegetable intake

**Figure 2:**
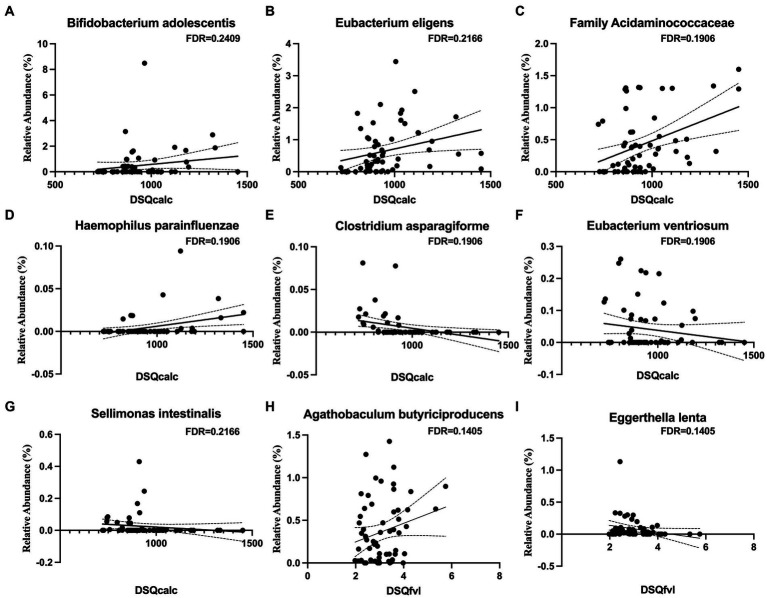
Microbial taxa associations with dietary calcium and vegetable intakes. Calcium intake is positively associated with **(A)** *Bifidobacterium adolescentis*, **(B)** *Eubacterium eligens*, **(C)** family *Acidaminococcaceae*, and **(D)** *Haemophilus parainfluenzae*, but negatively associated with **(E)** *Clostridium asparagiforme*, **(F)** *Eubacterium ventriosum*, and **(G)** *Sellimonas intestinalis*. Vegetable intake is positively associated with **(H)** *Agathobaculum butyriciproducens*, but negatively associated with **(I)** *Eggerthella lenta*.

We measured dietary intake over the previous month using the Dietary Screener Questionnaires (DSQ) in the National Health and Nutrition Examination Survey (NHANES) 2009-10. While there were no significant associations with fiber, whole grain, added sugar, dairy, significant associations existed with calcium intake and fruit and vegetable intake. An increased intake of calcium was associated a higher relative abundance of *Bifidobacterium adolescentis*, *Eubacterium eligens*, the family *Acidaminococcaceae*, and *Haemophilus parainfluenzae* (Figures 2A–D) and a lower abundance of *Clostridium asparagiforme*, *Eubacterium ventriosum*, and *Sellimonas intestinalis* (Figures 2E–G). An increased intake of vegetable was positively associated *Agathobaculum butyriciproducens* (Figure 2H), and negatively associated with *Eggerthella lenta* (Figure 2I).

### Gut microbiome associations with brain volume

**Figure 3:**
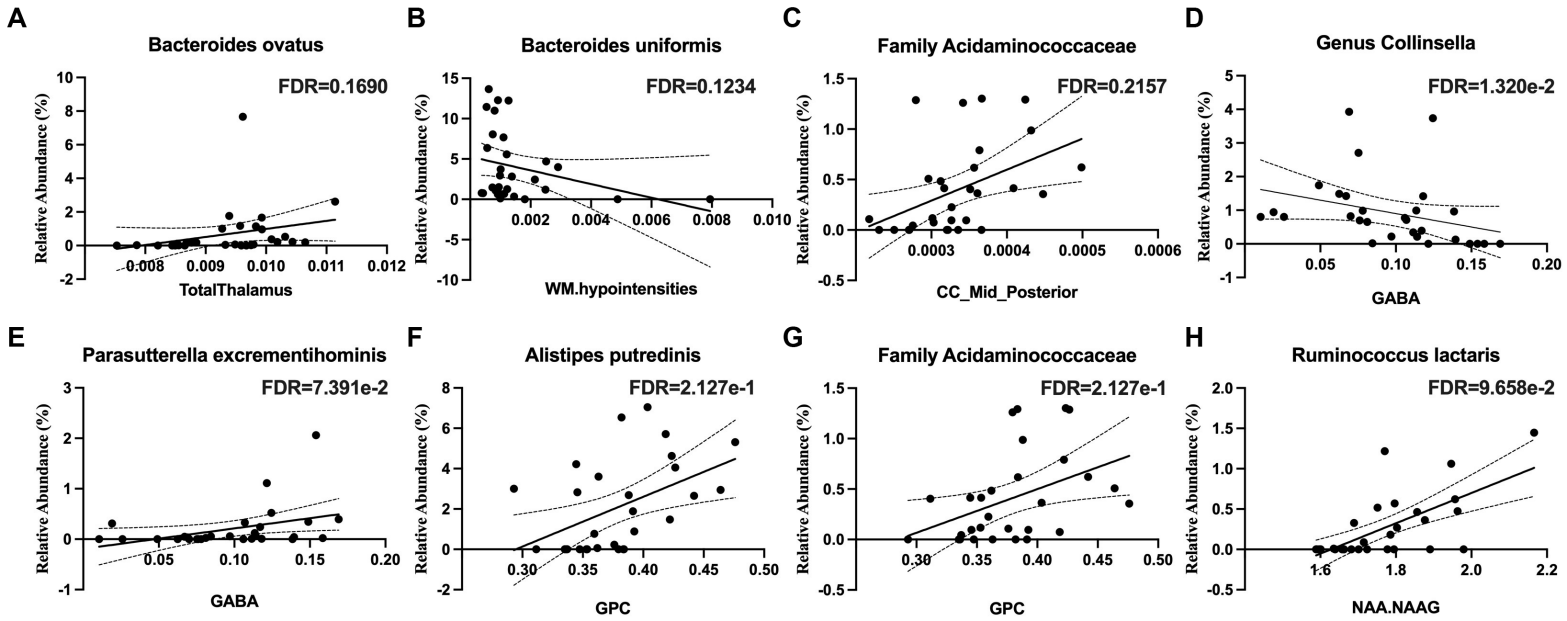
Microbial taxa associations with structural imaging and brain metabolites in the white matter of the corpus callosum. **(A)** The thalamus volume is positively associated with *Bacteroides ovatus* abundance. **(B)** White Matter (WM) hypointensities are positively associated with *Bacteroides uniformis* abundance. **(C)** The volume of the mid-posterior portion of the corpus callosum (CC_Mid_Posterior) is positively associated with the family *Acidaminococcaceae* abundance. **(D)** Gamma-aminobutyric acid (GABA) is negatively associated with the genus *Collinsella* and **(E)** positively associated with *Parasutterella excrementihominis*. **(F)** Glycerophosphorylcholine (GPC) is positively associated with *Alistipes putredinis* and **(G)** the family *Acidaminococcaceae*. **(H)** N-acetylaspartate (NAA) and N-acetylaspartylglutamate (NAAG) are positively associated with *Ruminococcus lactaris*.

The relative abundance of *Bacteroides ovatus* was positively associated with the thalamus volume (Figure 3A) and that of *Bacteroides uniformis* was negatively associated with white matter hypointensities (Figure 3B). The relative abundance of bacteria from the family *Acidaminococcaceae* was positively associated the volume of the mid posterior portion of the corpus callosum (Figure 3C).

### Gut microbiome associations with brain metabolites in white matter

The relative abundance *Collinsella aerofaciens* (*q* < 0.05) was negatively associated with gamma-aminobutyric acid (GABA) (Figure 3D) and that of *Parasutterella excrementihominis* was positively correlated with GABA (Figure 3E). The relative abundance of *Alistipes putredinis* and that of the family *Acidaminococcaceae* were positively correlated with glycerophosphocholine (GPC) (Figures 3F,G). The relative abundance of *Ruminococcus lactaris* was positively correlated with N-acetylaspartate and N-acetylaspartylglutamate (NAA + NAAG) (Figure 3H).

### Equol producers associated with white matter integrity (WMI)

**Figure 4:**
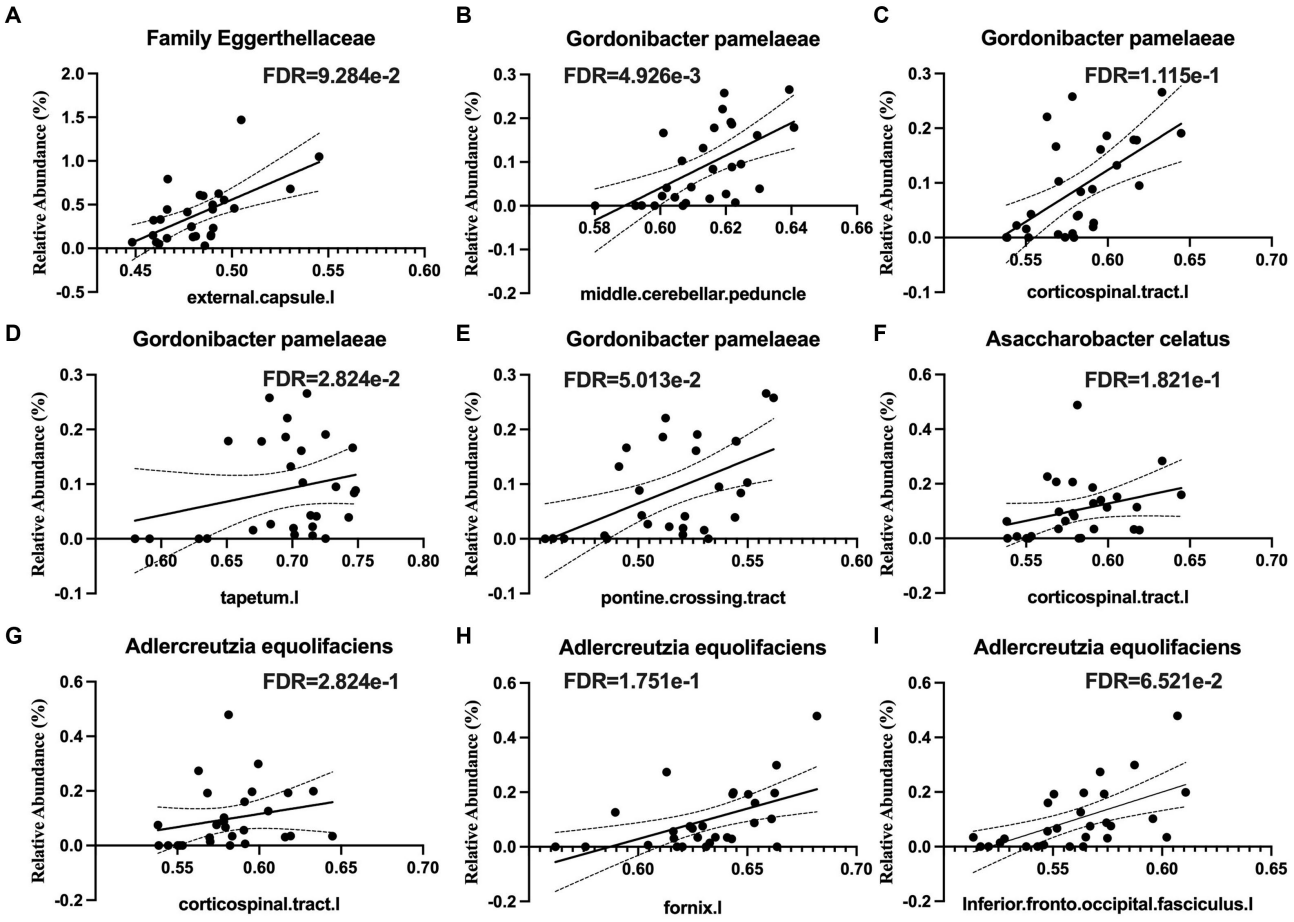
Species from the *Eggerthellaceae* family are positively correlated with white matter integrity (WMI) in language, memory, and limbic brain circuits. *Eggerthellaceae* spp. are positively associated with WMI in **(A)** the external capsule, **(B)** the middle cerebellar peduncle, **(C)** the corticospinal tract, **(D)** the tapetum, **(E)** the pontine crossing tract, **(F,G)** corticospinal tracts, **(H)** the fornix, and **(I)** the inferior fronto-occipital fasciculus.

**Figure 5:**
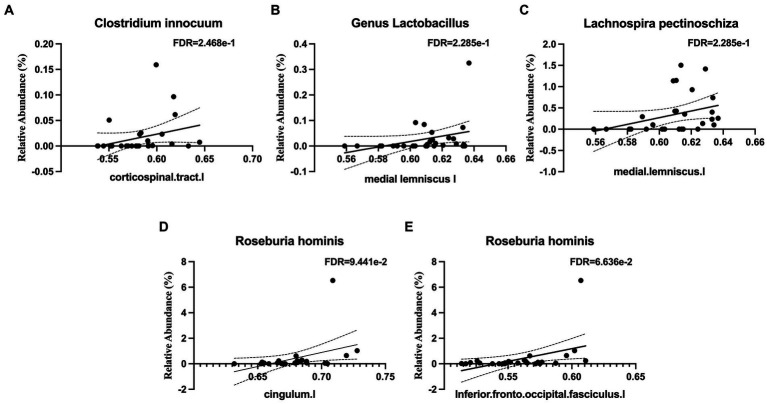
Notable microbial taxa associations with white matter integrity (WMI) in limbic and memory regions. **(A)** *Clostridium innocuum* is positively associated with WMI in the corticospinal tract. **(B)** The genus *Lactobacillus* is positively associated with WMI in the medial lemniscus. **(C)** *Lachnospira pectinoschiza* is positively associated with WMI in the medial lemniscus. *Roseburia hominis* is positively associated with WMI in **(D)** the cingulum and **(E)** the inferior fronto-occipital fasciculus.

Several taxa from the *Actinobacteria* were correlated with white matter integrity. These included the family *Eggerthellaceae* (Figure 4A), which was positively correlated with white matter tracts in the middle cerebellar peduncle and the left external capsule, which contains corticocortical association fibers. Specifically, the relative abundance of *Gordonibacter pamelaeae* (Figures 4B–E), *Asaccharobacter celatus* (Figure 4F), and *Adlercreutzia equolifaciens* (Figures 4G–I), were positively correlated with several tracts that connects brain regions responsible for language function and the limbic system. In the *Bacteroidetes* phylum, the relative abundance of *Coprobacter fastidiosus* was negatively correlated with the superior longitudinal fasciculus and that of *Bacteroides uniformis* was positively correlated with the tapetum. The *Firmicutes* phylum has several taxa that were positively correlated with white matter tracts, including *Clostridium innocuum* (Figure 5A), the genus *Lactobacillus* (Figure 5B), *Lachnospira pectinoschiza* (Figure 5C), *Roseburia hominis* (Figures 5D,E).

### Gut microbiome association with cerebral blood flow (CBF)

Table 2 shows the microbial taxa that were associated with CBF in various brain regions. The relative abundance of *Collinsella stercoris*, a bile-acid conjugator (Doden et al., 2021), was positively correlated with brain regions related language function and the limbic system, such as temporal cortex. The relative abundance of bacteria from the genus, part of the *Bacteroidetes* phylum and related to altered dopamine signaling through its synthetization of GABA (Strandwitz et al., 2019; Kramer et al., 2020), were negatively correlated with CBF in the basal ganglia regions and hippocampal regions, both implicated in learning and memory. In addition, the relative abundance of sulfate-reducing bacteria from the family *Desulfovibrionaceae*, known for inducing inflammatory responses and promoting atherosclerotic plaque formation, were negatively correlated with CBF in memory and learning areas.

**Table 2:** Microbial taxa associated with Cerebral Blood Flow (CBF).

Microbial taxa	Taxa key characteristics	Perfusion imaging feature	Coef	*Q* value
*Collinsella stercoris*	Bile-acid conjugator	Right banks of superior temporal sulcus	0.460	0.00409
		Total middle temporal cortex	0.340	0.1678
		Left pars triangularis	0.327	0.09401
		Left transverse temporal	0.339	0.2098
Genus *Parabacteroides*	Alter dopamine signaling	Right hippocampus	−0.753	0.00210
Family *Desulfovibrionaceae*	Inflammation inducer	Right entorhinal cortex	−0.449	0.1875

## Discussion

**Figure 6:**
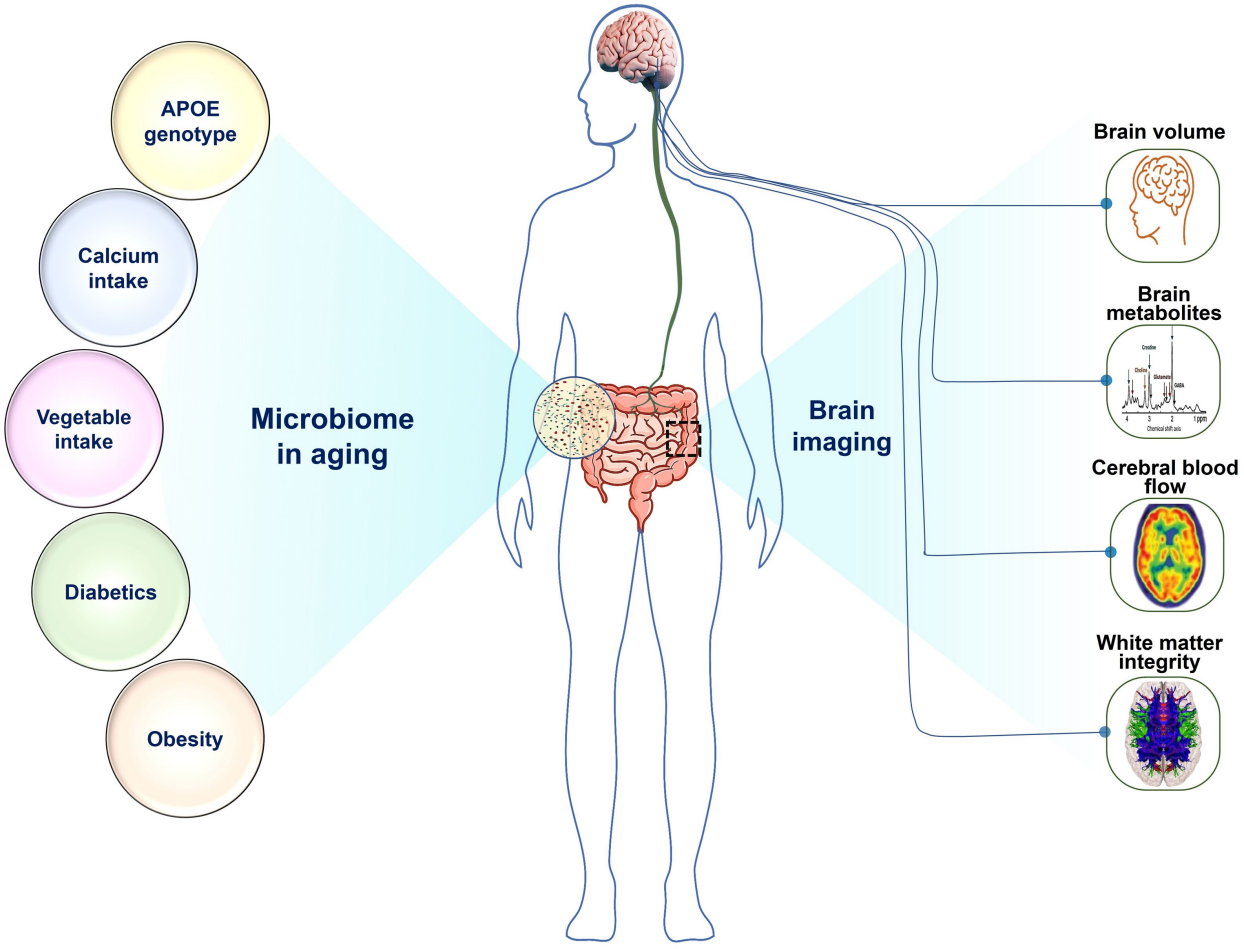
The relationship of gut microorganisms to various disease variables and brain function: Microbiome in aging is associated with (Left) APOE genotype, calcium intake, vegetable intake, diabetes, and obesity, as well as with (Right) brain imaging makers, including brain volume, brain metabolites, cerebral blood flow, and white matter integrity.

We conducted a study on the gut microbiome of 30 older adults who reside in the community. Figure 6 presents a summary of our findings, which indicate a strong association between microbiome composition in this group and several factors including APOE genotype, BMI, obesity, and diabetes, as well as brain imaging markers obtained from brain volume, CBF, metabolites, and WMI. Our analysis reveals that APOE2 and APOE4 carriers had a very distinctive microbiota composition are consistent with a number of studies examining gut microbiome in murine models (Weng et al., 2019; Parikh et al., 2020; Zajac et al., 2022). Specifically, the APOE4 genotype had relatively lower abundance of *Roseburia*, but higher in *Eubacterium eligens* and *Faecalibacterium prausnitzii*. On the other hand, the APOE2 genotype had higher levels of Lachnoclostridium species and lower levels of *Eubacterium eligens*, *Oscillibacter*, and *Faecalibacterium prausnitzii* (Chung et al., 2017). *Roseburia*, a well-known butyrate producer that is associated with a decreased risk of cognitive decline, was found to be decreased in the APOE4 genotype (Jin et al., 2021). This is an interesting finding as APOE2 is protective against Alzheimer’s disease (AD) while APOE4 increase the risk (Yamazaki et al., 2019).

While the functions of some species identified between APOE2 and APOE4 carriers in this study still need to be explored, our results align with existing literature, suggesting that APOE variants may shape the structure of the gut microbiome (Weng et al., 2019; Parikh et al., 2020; Zajac et al., 2022). In earlier work, we demonstrated that asymptomatic APOE4 carriers experience more pronounced gut dysbiosis than non-carriers, including those with APOE e3 alleles (APOE3), and prebiotic diet may mitigate the risk for AD by reducing the gut dysbiosis in the APOE4 mice (Hoffman et al., 2019; Yanckello et al., 2021). Other research has shown associations between APOE genotype differences and significant variations in the relative abundance of families like Prevotellaceae and Ruminococcaceae, as well as several butyrate-producing genera (Tran et al., 2019). A recent investigation even revealed that disruptions in gut microbiome composition can modify immune pathways, leading to the tauopathy underlying Alzheimer’s disease in an APOE genotype-dependent manner (Seo et al., 2023). These insights suggest that microbiome composition and diversity may play a substantial role in AD development, influenced by variations in APOE genetics. Moving forward, future studies should delve deeper into the differential effects of APOE-genotype dependent variations in the microbiome.

We found that obesity was inversely associated with *Akkermansia muciniphila*, which has previously been found to be decreased in obesity and is believed to be protective against metabolic disorders through its excretion of endocannabinoids that control inflammation, gut barrier function, and gut peptide secretion (Everard et al., 2013). Further, proinflammatory state was associated with higher BMI and diabetes. Diabetes was associated with increased levels of *E. coli* and *Parabacteroides goldsteinii*. While *E. coli* is a normal commensal gut bacteria, it has many pathologic variants that can cause disease (Leimbach et al., 2013), and increases in infectious *E. coli* have previously been reported in diabetes (Suri et al., 2009) and diabetes-related neurological disorders, such as stroke (Hammond et al., 2022a,b).

Our findings suggest that a healthy diet is strongly associated with a microbiota that produces SCFAs, leading to an anti-inflammatory state. We used the Dietary Screener Questionnaire from the National Health and Nutrition Examination Survey to measure effects of the diet on the microbiome. Interestingly, we observed an inverse relationship between calcium and *Clostridium asparagiforme*, a species that has been found to be reduced in smokers with hypertension (Wang et al., 2021). Conversely, we found that calcium was positively associated with *Acidaminococcaceae*, *Phascolarctobacterium* and *Eubacterium eligens*. The former two taxa are known to produce SCFAs (Wu et al., 2017), while *Eubacterium eligens* has anti-inflammatory effects on the host (Chung et al., 2017). Although the precise mechanisms underlying the effects of calcium on these microbial taxa remain unknown, previous research has shown that a high calcium diet increases *Firmicutes* species and levels of lactic, acetic, and butyric acid (Fuhren et al., 2021). We also observed a positive relationship between vegetable intake and *Agathobaculum butyriciproducens*, a butyrate producer, which has been shown to improve performance in behavioral tests, decrease Aβ plaque deposition and microglial activation APP/PS1 transgenic mice (Go et al., 2021). A negative effect with *Eggerthella lenta*, an infectious species, was found in our data (Gardiner et al., 2015; Go et al., 2021). It is interesting that fiber, whole grains, added sugars, dairy, and fruit intake did not have any significant associations with the microbial taxa in our data. Many studies have highlighted the beneficial effects of a vegetarian diet on the microbiome by limiting the abundance of inflammatory species and promoting SCFA-producing species (Tang et al., 2019; Sakkas et al., 2020). Other studies have investigated the effects of Western, Mediterranean, vegetarian, high carbohydrate, high fat, whole food, ultra-processed, and low-calorie sweetener diets on the microbiome (Dahl et al., 2020). Specific Dietary effects on the microbiome should be explored in future studies.

The results also imply that strengthening the immune barriers of the gut were associated with the volume of brain structures related to processing high levels of information. *Bacteroides ovatus* is related to the thalamus volume. The thalamus is an important relay station in the brain that communicates information from the spinal cord and cerebellum to the cerebral cortex (Sherman, 2007). *Bacteroides ovatus* is a commensal microbe that uses various carbohydrates and proteins as its fuel source (Fultz et al., 2021), is a prominent inducer of IgA (Yang et al., 2020), and promotes the reparative cytokine IL-22 (Ihekweazu et al., 2021), thereby stimulating epithelial recovery (Ihekweazu et al., 2019). The family *Acidaminococcaceae* is related to the volume of the mid posterior portion of the corpus callosum. The region is responsible for connecting portions of the parietal and temporal cortices on each side of the brain. This is consistent with a previous finding that *Acidaminococcaceae* is associated with memory performance (Khine et al., 2020).

Our results also indicated that microbiota associated with SCFAs production are highly associated with less vascular disease in the brain. *Bacteroides uniformis* correlates inversely with white matter hypointensities (WMH), which are a marker of vascular disease in the brain (Graff-Radford et al., 2019) and are often associated with cognitive impairment (Hu et al., 2021). *Bacteroides uniformis* prefers wheat bran extract as a fuel source and is a butyrate producer; it strengthens the first line of immune defense against unhealthy diets (Lopez-Almela et al., 2021) and improves glucose tolerance (Fabersani et al., 2021). This microbe has also been associated with increased dopamine transporters (Hartstra et al., 2020) and normalizing the brain reward response to reduce anxiety in rats (Agusti et al., 2021). Interestingly, the microbiota-derived phenylacetylglutamine has previously been associated with the amount of WMH in the brain (Yu et al., 2021).

Our data also suggests that gut microbiota that produce GABA, the main inhibitory neurotransmitter in the brain (Petroff, 2002), are highly associated with the amount of GABA present in the corpus callosum. *Collinsella aerofaciens* is negatively associated with GABA production, and *Parasutterella excrementihominis* is positively associated with GABA production. *Collinsella aerofaciens* is an obligate anaerobe (Bag et al., 2017; Qin et al., 2019) and a proinflammatory species associated with Crohn’s disease (Joossens et al., 2011; Kalinkovich and Livshits, 2019). *Parasutterella excrementihominis* is strictly anaerobic (Nagai et al., 2009) and has been associated with impaired GI health (Fart et al., 2020). While *Collinsella aerofaciens* and *Parasutterella excrementihominis* have not previously been identified as GABA producers or consumers, *Bacteroides* has been identified as a dominant GABA producer and *Pseudomonas* as a prominent GABA consumer (Strandwitz et al., 2019).

It was observed that microbial species *Ruminococcus lactaris*, a producer of SCFAs, is associated with the brain metabolites related to mitochondrial function and cognition. NAA correlates with neuronal mitochondrial function and survival (Paslakis et al., 2014), and NAAG has procognitive properties (Neale and Olszewski, 2019). *Ruminococcus lactaris* is a butyrate producer (Sato et al., 2021) and is negatively correlated with the inflammatory cytokine IL-8 (Shintouo et al., 2020). A previous group found a link between *Ruminococcus* and NAA thought to be mediated by *Ruminococcus* decreasing cortisol, which impacts brain NAA (Mudd et al., 2017).

Our analysis indicates a positive correlation between *Collinsella stercoris*, a producer for conjugated bile acid, with CBF. Previous research has shown that the conjugated bile acid Tauroursodeoxycholic acid (TUDCA) can alleviate early brain injury by reversing cerebrovascular dysfunction and ER-stress-mediated apoptosis (Chen et al., 2020). TUDCA was found to enhance neurological function and treated SAH-related cerebrovascular dysfunction by increasing cerebral cortical perfusion and decreasing blood–brain barrier permeability. These reports are in line with our findings that presence of *Collinsella stercoris* may be beneficial for maintaining CBF. We further showed that microbiota related to equol producers are highly associated with WMI that connecting brain regions associated with language and memory. The superior temporal sulcus is commonly referred to as Wernicke’s area and is a central area for speech recognition and processing (Nourski et al., 2021). The left pars triangularis is commonly referred to as Broca’s area and is a central area for speech production (Foundas et al., 1996) Members of the family *Eggerthellaceae* are able to convert the isoflavone daidzein (a soy product) into equol, an estrogen (Soukup et al., 2021). Specific members of this family, including *Adlercreutzia equolifaciens*, *Asaccharobacter celatus*, and *Gordonibacter pamelaeae* all have negative associations with these same areas responsible for language circuits, in addition to areas of the brain responsible for memory, learning, and reward processing. Equol supplementation has been shown to improve long and short-term memory in rats by increasing brain antioxidant activity and improving blood pressure (Liu and Tsai, 2016; Caliskan et al., 2021).

Collectively, the findings in the present study indicated that microbiome composition in aging may play an important role in determining brain metabolism and integrity, and *APOE* status could also be a key player. This is in line with studies showing that metabolism may play a more critical role in driving neurodegeneration than amyloid beta plaques and tau tangles in the progression of Alzheimer’s disease (AD) (Hammond et al., 2020; Hammond and Lin, 2022), and that *APOE4* carriers and non-carriers develop AD through different metabolic pathways (Hammond et al., 2021). Therefore, it will be critical for to identify interventions that can promote healthy microbiome in aging, taking into consideration of APOE genotype, to protect brain function in aging and potentially mitigate AD risk for APOE4 carriers. This is in good agreement with our recent studies showing that supplementing a diet with the prebiotic inulin, a fermentable prebiotic fiber, can positively impact gut microbiome composition, boost the production of SCFAs, enhance mitochondrial function, and decrease neuroinflammation in young, asymptomatic *APOE4* mice (Hoffman et al., 2019). Our results also demonstrate that these effects of dietary inulin supplementation revealed an *APOE* genotype-dependent response (Yanckello et al., 2021). These findings are also consistent with what we observed in the current study that higher fiber (vegetable) intake can improve microbiome in aging. Our study further provides other potential interventions in the future, such as equol or butyrate supplementation, as well as probiotic interventions with butyrate and equol producers like *Roseburia*, which have shown promise as easily implemented, inexpensive tools to optimize healthy brain aging.

While this study provides further understanding between gut microbiome and brain health in the aging population, it has many limitations. The observational nature of this study helps us to make many associations between gut bacteria and markers of brain health on imaging, but we are unable to determine causation. Additionally, due to the pandemic, we were only able to recruit 30 participants for the study. The relatively small sample size for a human study with a heterogeneous population potentially leaves the study underpowered to detect smaller associations and changes in the microbiome. Future work should utilize a larger sample size to allow adjusting for potential confounders and to identify which demographic factors (e.g., BMI, gender, age, APOE genotype) most strongly associate with the composition of the microbiome. Future work should investigate the long-term nature of the gut microbiome in this population. Future studies can also further include other more advanced MRI methods to measure brain oxygen metabolism or perivascular space, which will provide further insight for brain metabolism and inflammation, respectively (Lin et al., 2008, 2010; Feldman et al., 2018; Langan et al., 2022).

In summary, we demonstrated the significant correlations of microbiome in older adults with their brain imaging markers, APOE genotypes, calcium intake, vegetable intakes, diabetes, and obesity. The findings could have important implications in the future for promoting brain health through microbiome modulation and developing precision nutrition interventions to optimize healthy brain aging older adults.

## Data availability statement

The datasets presented in this article are not publicly available due to national legislation that restricts the deposition of human data. Requests to access the datasets should be directed to A-LL ai-ling.lin@health.missouri.edu.

## Ethics statement

The studies involving humans were approved by Institutional Review Board at the University of Kentucky. The studies were conducted in accordance with the local legislation and institutional requirements. The participants provided their written informed consent to participate in this study.

## Author contributions

TH, AS, and A-LL designed the research. TH, SG, GC, AC, and A-LL conducted the research. TH, AC, YJ, XX, and AS performed the statistical analysis. TH, SG, GC, YJ, SH, AC, CA, XX, AF, AS, PB, and A-LL wrote the manuscript. TH, SG, AS, and A-LL had primary responsibility for final content. All authors contributed to the article and approved the submitted version.

## Funding

This research was supported by grants from NIH/NIA, NIH/ODS, and American Federation for Aging Research to A-LL (R01AG054459 and RF1AG062480) and TH (T32AG057461).

## Conflict of interest

The authors declare that the research was conducted in the absence of any commercial or financial relationships that could be construed as a potential conflict of interest.

## Publisher’s note

All claims expressed in this article are solely those of the authors and do not necessarily represent those of their affiliated organizations, or those of the publisher, the editors and the reviewers. Any product that may be evaluated in this article, or claim that may be made by its manufacturer, is not guaranteed or endorsed by the publisher.

## Footnotes

^1^ https://surfer.nmr.mgh.harvard.edu/fswiki/RcbfProc
